# Systemic inflammatory response in colorectal cancer is associated with tumour mismatch repair and impaired survival

**DOI:** 10.1038/s41598-024-80803-6

**Published:** 2024-11-29

**Authors:** Mats Hjortborg, Sofia Edin, Camilla Böckelman, Tuomas Kaprio, Xingru Li, Ioannis Gkekas, Jaana Hagström, Karin Strigård, Caj Haglund, Ulf Gunnarsson, Richard Palmqvist

**Affiliations:** 1 Department of Diagnostics and Intervention, Umeå University, Umeå, Sweden; 2 Department of Medical Biosciences, Pathology, Umeå University, Umeå, Sweden; 3 Department of Gastrointestinal Surgery, University of Helsinki, Helsinki University Hospital, Helsinki, Finland; 4 Department of Pathology, Department of Oral Pathology and Radiology, University of Helsinki, University of Turku, Helsinki, Finland

**Keywords:** Colorectal cancer, Systemic inflammatory response, Mismatch repair, Immunity, Prognosis, Cancer microenvironment, Colorectal cancer, Tumour immunology

## Abstract

The systemic inflammatory response (SIR), defined as elevated levels of circulating C-reactive protein (CRP), is an important predictor of impaired survival in colorectal cancer. The aim of this study was to explore the prognostic role of SIR and its association with tumour mismatch repair status and the immune response. Immune activity profiles of mononuclear cells isolated from CRC tissues and blood in the U-CAN exploration cohort (*n* = 69), were analysed by flow cytometry. In the U-CAN validation cohort (*n* = 257), T-helper cells (T-bet^+^), cytotoxic T cells (CD8^+^), regulatory T cells (FoxP3^+^), B cells (CD20^+^), and macrophages (CD68^+^) were analysed by multispectral imaging. Microsatellite instability was determined using five mononucleotide-repeat microsatellite markers. Patients with high CRP levels (> 10 mg/l) were significantly more often diagnosed with high-grade tumours and tumours exhibiting microsatellite instability. However, some patients with high CRP levels were found to have microsatellite-stable tumours. Furthermore, high CRP levels were associated with specific tumour immune traits including an augmented macrophage response and were significantly linked to poorer cancer-specific survival, particularly in patients with microsatellite-stable tumours. In conclusion, our findings suggest an interplay between SIR and mismatch repair status in CRC prognosis which needs to be further explored.

**Supplementary Information:**

The online version contains supplementary material available at 10.1038/s41598-024-80803-6.

## Introduction

Colorectal cancer (CRC) is the third most common form of cancer worldwide. The overall 5-year survival rate for patients with CRC has now reached approximately 65%^1^. Tumour stage at diagnosis is the main determinant of survival, but CRC is a heterogeneous disease with different molecular characteristics including genetic and epigenetic changes. Staging, based on the pathologist’s evaluation of the resected tumour and on preoperative imaging findings, provides some indication of prognosis and is used for postoperative stratification of patients. A weakness of the TNM classification is the inability to discriminate between tumours within the same stage that have biological markers indicating a favourable or poor prognosis. This leads to imprecise outcome prediction, particularly in Stages II and III. Nearly 20% of Stage II patients die of a recurrence^2^. The accuracy of survival prediction thus needs to be improved.

There is increasing evidence that the host’s inflammatory response plays an important role in tumour progression and thereby prognosis^3^. Inflammatory biomarker patterns arising from the tumour microenvironment play a large role in patient outcome^4^. The systemic inflammatory response (SIR), defined as elevated levels of circulating C-reactive protein (CRP), is an independent risk factor for impaired survival^5–8^. A stage by stage relationship between SIR and impaired long-term survival was described by our group in a pioneer proof-of-concept study^6^, and has since been confirmed by several other groups^8–10^. We confirmed this association in a subsequent study where SIR was shown to be a much better predictor of poor survival than tumour size in patients with CRC liver metastases^7^.

According to the Consensus Molecular Subtypes (CMS), CRC tumours are divided into four groups (CMS 1–4) based on their transcriptome^11,12^. These four tumour subtypes have different epigenomic, transcriptomic, microenvironmental, genetic, and clinical characteristics. CMS1 tumours are characterised by a proximal location, high immunogenicity, high *BRAF*-mutation rate, a CpG island methylator phenotype (CIMP), and display microsatellite instability (MSI). CMS2 tumours are characterised by epithelial differentiation and are mainly left-sided. CMS3 tumours are characterised by a higher CIMP status and high *KRAS-*mutation rate, while CMS4 tumours are characterised by a distal location, CIMP negativity, and are often microsatellite stable (MSS).

CMS1/MSI tumours are of special interest due to impairment of the DNA mismatch repair (MMR) system, leading to high immunogenicity and infiltration by lymphocytes. There is also clinical evidence that patients with early stage MSI tumours have a better prognosis compared to patients with MSS tumours^13–15^.

The local tumour response is known to be an important prognosis factor in CRC. Tumours highly infiltrated by lymphocytes tend to have a better outcome^3^, and in some cases this predicts the tumour’s response to chemotherapy and immune checkpoint blockade^16^.

In this study, we investigated the association of SIR with tumour molecular subtypes and the anti-tumour immune response in two CRC patient cohorts, aiming to further explore the prognostic role of SIR.

## Results

### High CRP levels were associated with tumours of high-grade and a right-sided location

**Fig. 1:**
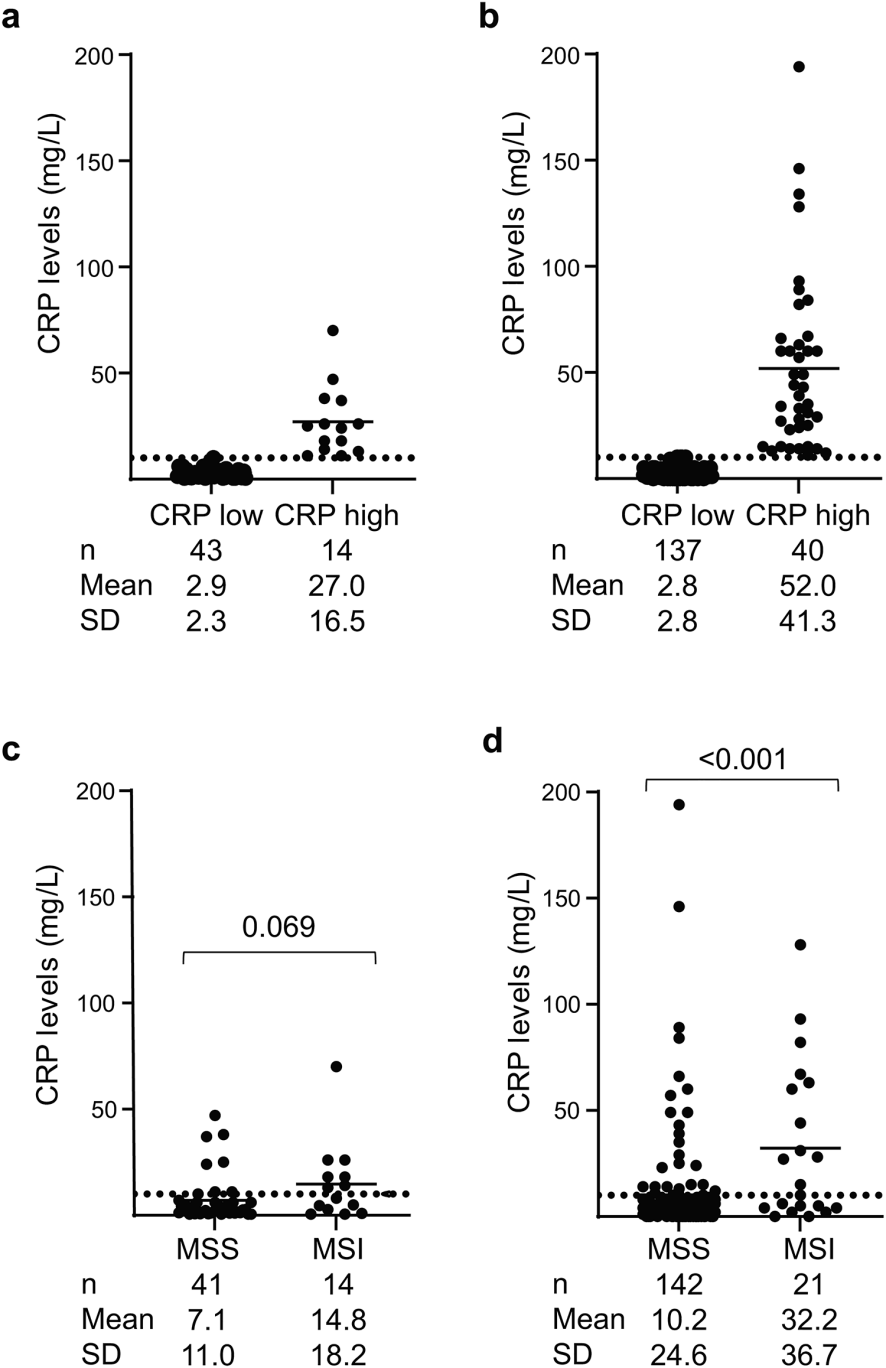
The distribution of CRP levels in patients with CRC. Top panel, scatter plots displaying the levels of CRP in groups of CRC patients defined by low or high CRP levels in (**A**) patients from the U-CAN exploration cohort, or (**B**) patients from the U-CAN validation cohort. Bottom panel, scatter plots displaying the levels of CRP in groups of CRC patients with MSI or MSS tumours in (**C**) patients from the U-CAN exploration cohort, or (**D**) patients from the U-CAN validation cohort. Horizontal lines indicate mean relative levels (mg/l). Dotted lines indicate CRP cut-off (10 mg/l) used to define low and high levels of CRP.

Preoperative CRP levels were collected from patients included in the U-CAN exploration cohort (UIP-CRC, *n* = 69) and the U-CAN validation cohort (*n* = 257). Patients were divided into groups with low CRP (≤ 10 mg/l) and high CRP (> 10 mg/l) levels (Fig. 1A and B), where a high CRP level with no known underlying cause was considered to be due to SIR.

Associations between CRP and clinical and pathologic characteristics are seen in Table 1. Twenty-five per cent of patients from the U-CAN exploration cohort and 23% of patients from the U-CAN validation cohort had high CRP levels. Significantly more of these patients had a high-grade tumour compared to those with low CRP levels (Table 1). An association between high CRP levels and a right-sided colon cancer location was also observed, reaching significance in the U-CAN validation cohort. A significant association was also seen between high CRP levels and a mucinous tumour type in the U-CAN validation cohort (Table 1).

**Table 1:** Associations of CRP with clinical and pathological characteristics.

	U-CAN exploration (*n*=69)			U-CAN validation (*n*=257)		
	CRP Low	CRP High	*P* value	CRP Low	CRP High	*P* value
Frequency, n (%)	43 (75.4)	14 (24.6)		137 (77.4)	40 (22.6)	
Age, n (%)			0.314			0.198
≤ 59	6 (75.0)	2 (25.0)		26 (83.9)	5 (16.1)	
60–69	9 (90.0)	1 (10.0)		55 (83.3)	11 (16.7)	
70–79	20 (80.0)	5 (20.0)		39 (70.9)	16 (29.1)	
≥ 80	8 (57.1)	6 (42.9)		17 (68.0)	8 (32.0)	
Sex, n (%)			0.548			0.589
Male	23 (71.9)	9 (28.1)		74 (75.5)	24 (24.5)	
Female	20 (80.0)	5 (20.0)		63 (79.7)	16 (20.3)	
Tumour location, n (%)			0.139			<0.001
Right colon	22 (71.0)	9 (29.0)		27 (57.4)	20 (42.6)	
Left colon	7 (63.6)	4 (36.4)		29 (78.4)	8 (21.6)	
Rectum	14 (93.3)	1 (6.7)		81 (87.1)	12 (12.9)	
Stage, n (%)			0.418			0.118
I	9 (81.8)	2 (18.2)		29 (87.9)	4 (12.1)	
II	17 (85.0)	3 (15.0)		52 (83.9)	10 (16.1)	
III	14 (63.6)	8 (36.4)		32 (76.2)	10 (23.8)	
IV	3 (75.0)	1 (25.0)		21 (65.6)	11 (34.4)	
Tumour grade, n (%)			0.002			0.002
Low grade	35 (87.5)	5 (12.5)		114 (87.0)	17 (13.0)	
High grade	8 (47.1)	9 (52.9)		11 (55.0)	9 (45.0)	
Tumour type, n (%)			1.00			<0.001
Non-mucinous	38 (76.0)	12 (24.0)		118 (86.8)	18 (13.2)	
Mucinous	5 (71.4)	2 (28.6)		9 (50.0)	9 (50.0)	
Preoperative RT, n (%)			NA			0.006
No	43 (75.4)	14 (24.6)		88 (71.5)	35 (28.5)	
Yes	0 (0.0)	0 (0.0)		49 (90.7)	5 (9.3)	

Fischer´s exact test was used for comparisons. The following variables were missing in the U-CAN validation cohort: tumour stage, 8 cases; tumour grade, 26 cases; and tumour type, 23 cases.

### High CRP levels were associated with MSI subtype tumours

Associations between high preoperative CRP levels and molecular characteristics of the tumour were also explored (Table 2). There was a significant association between high CRP levels and *BRAF*-mutated tumours in the U-CAN validation cohort. No association was seen between high CRP levels and *KRAS*-mutated tumours. In both cohorts, a high CRP level was significantly linked to tumours of the MSI subtype. Stratifying CRP analyses according to MMR status by *BRAF*-mutation further showed that the association with high CRP levels was mainly linked to MSI. In the UCAN-exploration cohort, there was no significant association between CMS subtypes 1–4 and high CRP levels (Table 2).

**Table 2:** Associations of CRP with tumour molecular characteristics.

	U-CAN exploration (*n*=69)			U-CAN validation (*n*=257)		
	CRP Low	CRP High	*P* value	CRP Low	CRP High	*P* value
Frequency, n (%)	43 (75.4)	14 (24.6)		137 (77.4)	40 (22.6)	
*KRAS*-status, n (%)			0.705			0.546
Wild-type	29 (70.7)	12 (29.3)		80 (76.2)	25 (23.8)	
Mutant	9 (81.8)	2 (18.2)		44 (81.5)	10 (18.5)	
*BRAF*-status, n (%)			0.216			0.025
Wild-type	29 (80.6)	7 (19.4)		109 (82.0)	24 (18.0)	
Mutant	13 (65.0)	7 (35.0)		18 (62.1)	11 (37.9)	
*BRAF/KRAS*-status, n (%)			0.615			0.071
Wild-type/Wild-type	16 (76.2)	5 (23.8)		63 (81.8)	14 (18.2)	
*KRAS*-mutant	9 (81,8)	2 (18.2)		44 (81.5)	10 (18.5)	
*BRAF*-mutant	13 (65.0)	7 (35.0)		17 (60.7)	11 (39.3)	
MMR status, n (%)			0.029			<0.001
MSS	34 (82.9)	7 (17.1)		118 (83.1)	24 (16.9)	
MSI	7 (50.0)	7 (50.0)		10 (47.6)	11 (52.4)	
MSI/*BRAF-*status, n(%)			0.062			0.003
MSS/*BRAF* wild-type	27 (84.4)	5 (15.6)		106 (83.5)	21 (16.5)	
MSS/*BRAF*-mutant	7 (77.8)	2 (22.2)		11 (78.6)	3 (21.4)	
MSI/*BRAF* wild-type	1 (33.3)	2 (66.7)		3 (50.0)	3 (50.0)	
MSI/*BRAF* mutant	6 (54.5)	5 (45.5)		7 (46.7)	8 (53.3)	
CMS subtype, n (%)			0.077			
CMS1	9 (52.9)	8 (47.1)				
CMS2	17 (85.0)	3 (15.0)				
CMS3	2 (66.7)	1 (33.3)				
CMS4	4 (100.0)	0 (0.0)				

Fischer´s exact test was used for comparisons. The following variables were missing in the U-CAN exploration cohort: *KRAS*-status, 5 cases; *BRAF*-status, 1 case; *KRAS/BRAF*-status, 5 cases; MSI status, 2 cases; MSI/*BRAF*-status, 2 cases; and CMS status, 13 cases. Missing cases in the U-CAN validation cohort were: *KRAS-* status, 18 cases; *BRAF*-status, 15 cases; *KRAS/BRAF*-status; 18 cases, MSI status, 14 cases; and MSI/*BRAF* status, 15 cases.

Using CRP levels as a continuous variable and comparing these to tumour MMR status, higher CRP levels were associated with MSI tumours compared to MSS tumours (Fig. 1C and D), reaching significance in the U-CAN validation cohort (Fig. 1D). However, some patients with an MSS tumour had a high CRP level (Fig. 1D).

### High CRP levels were associated with increased tumour and systemic immune markers

**Fig. 2:**
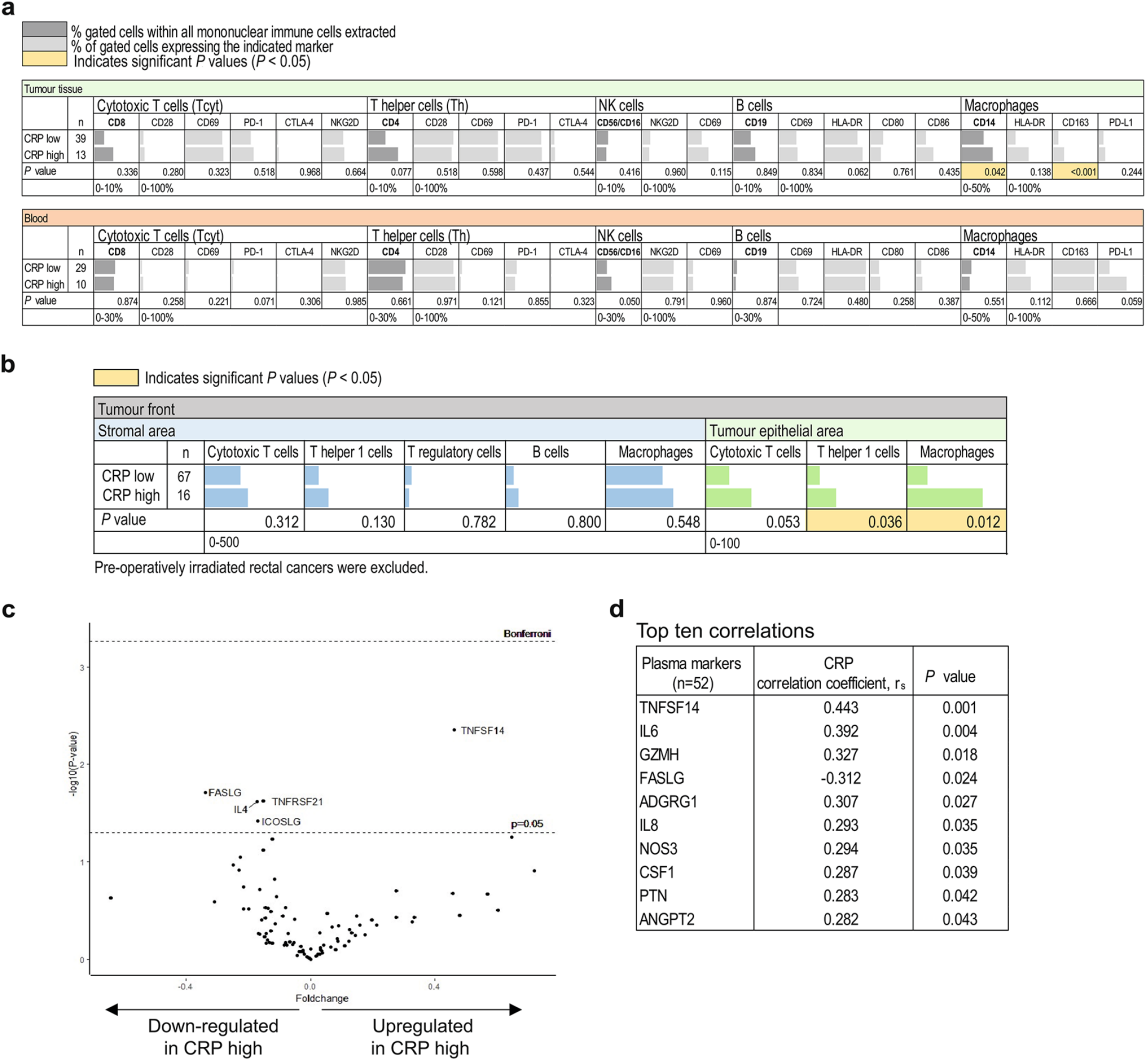
Relations of CRP to immune parameters in tumour tissue and blood of CRC patients. (A) Flow cytometry analyses of immune parameters in groups of patients with low or high CRP. Shown is the median percentage of gated cell populations within mononuclear immune cells (in dark grey) isolated from tumour tissue or blood of patients with colorectal cancer in the U-CAN exploration cohort, as indicated, and percentage of these gated cells expressing the indicated markers (in light grey). (B) Immune cell tumour infiltration was analysed in patients from the U-CAN validation cohort using multiplex immunohistochemistry and multispectral imaging. Shown are median numbers of infiltrating cells/mm^2^ stromal or tumour epithelial area within the tumour border in patients with low or high levels of CRP. Preoperatively irradiated rectal cancers were excluded. (C) Volcano plot displaying the differential distribution of plasma factors in high CRP compared to low CRP CRC patients from the U-CAN exploration cohort as analysed by the OLINK Immuno-oncology panel. (D) Shown are top ten correlations between plasma marker levels and CRP levels in CRC patients from the U-CAN exploration cohort. Abbreviation: r_s_, Spearman´s rank correlation coefficient.

The association between CRP level and immune markers in tumour tissue and in blood was investigated. In the U-CAN exploration cohort, flow cytometry analyses of immune markers in tumour tissue revealed a slight but non-significant increase in the fractions of cytotoxic T-cells (CD8^+^) and T-helper cells (CD4^+^) in patients with a high CRP level compared to those with a low CRP level, as well as significantly higher fractions of macrophages (CD14^+^), expressing both M1 (HLA-DR^+^) and M2 markers (CD163^+^) (Fig. 2A). Results from parallel analyses of immune cells in blood showed no significant associations (Fig. 2A). Immune cell infiltration was further explored in the U-CAN validation cohort using multiplex immunohistochemistry and multispectral imaging to detect cytotoxic T cells (CD8^+^), T helper 1 cells (T-bet^+^), T regulatory cells (FoxP3^+^), B cells (CD20^+^), and macrophages (CD68^+^) at the tumour front. Significantly higher intra-epithelial infiltration of T-bet^+^ cells and macrophages were seen in tumours of patients with a high CRP level compared to those with a low CRP level (Fig. 2B). Similar findings were seen for intra-epithelial infiltration by cytotoxic T cells (Fig. 2B).

The relationship between CRP level and systemic immune markers was analysed in the U-CAN exploration cohort. Systemic markers in plasma were analysed using the OLINK Immuno-oncology panel that identifies 92 different proteins. Patients with a high CRP level showed upregulation of TNFSF14 and downregulation of FASLG, TNFRSF21, IL4, and ICOSLG, compared to patients with a low CRP level (Fig. 2C). Furthermore, when analysed as a continuous variable, increasing CRP levels were significantly correlated to increased levels of several immune markers including TNFSF14, IL6, GZMH, IL8 and CSF1, and decreased levels of FASLG (Fig. 2D).

### High CRP levels were associated with decreased patient survival

**Fig. 3:**
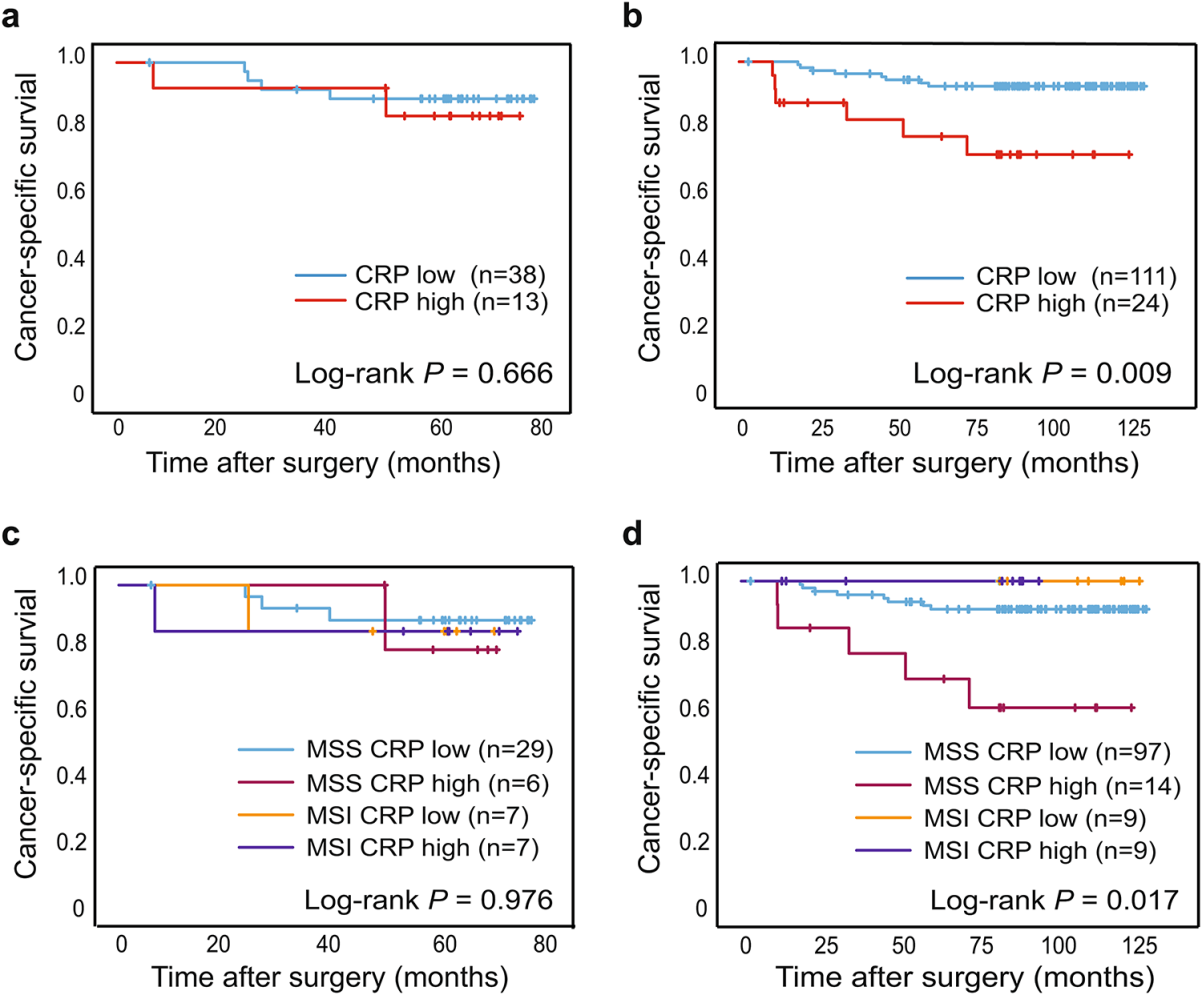
The prognostic importance of CRP levels in Stage I-III CRC patients. Shown are Kaplan-Meier plots of cancer-specific survival in patients with low or high CRP levels in all CRC patients (A, B), or stratified by tumour MSI status (C, D) in patients from the U-CAN exploration cohort (A, C) and in patients from the U-CAN validation cohort (B, D). Log-rank tests were used to calculate differences in 5-year survival between groups.

The association between CRP level and cancer-specific survival was analysed in both patient cohorts. Kaplan-Meier curves were created for Stage I-III CRC patients with either low or high CRP levels. Patients with a high CRP level in the U-CAN validation cohort were found to have a significantly reduced 5-year cancer-specific patient survival compared to those with a low CRP level (Fig. 3B). When stratifying for MSI status in the U-CAN validation cohort, a high CRP level indicating poor prognosis was mainly seen in patients with an MSS tumour (Fig. 3D).

The negative prognostic value of CRP was maintained in a multivariable Cox proportional hazards model with age, sex, tumour site, and stage as variables (Supplementary Table S1). The model was statistically underpowered making the addition of further variables such as MMR status to the model unsuitable.

## Discussion

Associations were found between high CRP levels (SIR) and MSI tumours as well as an increased local anti-tumour immune response. Most patients with a high CRP level had a poor survival rate, especially those patients with an MSS tumour. This emphasizes the fact that SIR and MSI should be treated as two separate entities in the prognostic assessment of CRC.

Approximately 15% of CRC patients have an MSI tumour resulting from a defect in the MMR system. MSI is observed more frequently in women and in CRCs located proximally^17^. These tumours also exhibit greater lymphocyte infiltration rate, poor differentiation, and a mucinous cell type^18,19^. CRCs with MSI are usually large primary tumours that tend not to spread to lymph nodes and metastasise^20^. According to Gkekas et al.^14^, analysis of time to progression and cancer-specific-survival showed that deficient MMR is a strong positive prognostic factor in colon cancer Stage II.

In this study, higher CRP levels were observed in patients with a right-sided or an MSI tumour, as previously reported in several papers^6,21–24^. However, despite the strong association between high CRP levels and MSI tumours, some patients with an MSS tumour had the highest CRP levels, an observation previously made by our group^25^. This finding indicates that there could be a subgroup within MSS tumours with unique characteristics. Interestingly, we have in a previous study shown that a subgroup of MSS tumours share immune characteristics with MSI tumours^26^. The found increased immunogenicity of some MSS tumours has been corroborated by other studies, including but not restricted to *POLE-*mutated tumours^27–30^. Further investigations in larger patient cohorts are needed to understand the biology behind CRP-high MSS tumors. In line with the high immunogenicity of MSI tumours, we also saw significant associations between high CRP levels and a high degree of tumour infiltration by macrophages in both the exploration and the validation cohort measured in two different ways, emphasizing the consistency of these findings. In the validation cohort, intra-epithelial infiltration of the tumour by T-cells, especially T-helper 1 cells, was associated with high CRP levels. These findings have been corroborated by Kostner et al., in a study where systemic inflammation was linked to increased features of tumour myeloid characteristics including increased infiltration by macrophages^31^. Immune cell infiltration of the tumour is a strong positive prognostic factor in CRC, where infiltration of T cell- and macrophage subsets has been found to be associated with improved survival^3,32^. The prognostic relevance of local adaptive immune responses supports the use of quantification of immune infiltration of the tumour when predicting the outcome of CRC disease.

On the other hand, a high CRP level was not found to be associated with an increase in the systemic cellular immune response. There was, however, an association between CRP and other non-cellular systemic immune markers, including IL6. A positive association between systemic CRP levels and IL6 has previously been shown in CRC patients^33,34^. IL6 is a pro-inflammatory cytokine that has a vital role in tumour progression through growth-promotion, anti-apoptotic activity, and modulation of the immune response^35^. A high systemic IL-6 level has also been shown to be an independent negative prognostic factor in CRC^36,37^, in line with the observations reported in this paper.

It is well known that both *KRAS*- and *BRAF*-mutations, regardless of MSI status, are associated with a poor prognosis^38,39^. *BRAF*-mutation is associated with MSI tumours, though it is also seen in MSS tumours^40^. *BRAF*-mutated MSS tumours appear to have the worst prognosis^41^. In the UCAN-validation cohort there was a significant association between high CRP levels and *BRAF*-mutated tumours, but a stratified analysis of CRP and MMR revealed a stronger association between CRP and MSI than with *BRAF-*mutation. No significant association was found between high CRP levels and *KRAS*-mutated tumours in this study. However, *KRAS-*mutation has previously been linked to lower CRP levels^42^.

In the U-CAN validation cohort, a high CRP level was strongly associated with high tumour grade, as reported previously^6^, and with mucinous tumour type. This emphasizes the presence of specific biological tumour characteristics in tumours exhibiting SIR.

A high CRP level was identified as a strong negative prognostic factor for survival in Stage I-III CRC patients in the U-CAN validation cohort. Previous studies from our group^6,25^, along with those from others^5–8,43^support the notion that a high preoperative CRP level is strongly associated with poorer cancer-specific survival in patients with colorectal cancer. In this study, our findings further suggest that the negative prognostic role of SIR in CRC was mainly seen in patients with an MSS tumour. These findings are supported by He et al., who also suggested that the prognostic role of systemic inflammatory factors in CRC is restricted to patients with an MSS tumour^21^. However, contradictory findings have also been reported^22,23^.

The reason behind the possible association between SIR and poor prognosis specific to patients with an MSS tumour is not fully understood. The association between SIR and MSI tumours as well as an increased local anti-tumour immune response could perhaps improve our assessment of prognosis in cases of MSI tumour with SIR. In a study by Mori et al., patients with a combination of high CRP level and low local anti-tumour immune infiltration were shown to have a poorer prognosis than patients with only one or the other^44^. Nevertheless, our findings suggest an association between SIR and MMR status when assessing CRC prognosis that needs further exploration.

A strength of this study is that it is based on two cohorts with an inclusion period of seven years (2010–2017). The U-CAN exploration cohort, though small, provides detailed analyses of immune profiles from both the tumour and the cellular and non-cellular compartments of blood, along with descriptive analyses of molecular subtypes. This enabled more precise analyses of immune mechanisms behind SIR. A weakness of the U-CAN exploration cohort was the limited number of patients. The findings should therefore be interpreted with some caution from a statistical point of view. The main findings, however, were supported in the larger U-CAN validation cohort. A weakness of the validation cohort, however, was that multivariable survival analyses were restricted due to limited sample size. This underlines the need for further larger studies to establish the impact of MMR status on the prognostic importance of SIR.

We report an association between elevated preoperative CRP levels (SIR) and MMR status in CRC. Furthermore, the negative prognostic impact of SIR was found to be dependent on tumour MMR status and highly significant in patients with an MSS tumour. Further studies are needed to establish the importance of SIR and MMR status when assessing prognosis in CRC.

## Materials and methods

### Study cohorts

This study was based on two patient cohorts from the Uppsala-Umeå Comprehensive Cancer Consortium (U-CAN) project^45^. Within U-CAN, blood, formalin-fixed paraffin-embedded (FFPE) tissue, fresh frozen tissue, faeces, radiology data, and clinical data have been collected over time from the CRC patients enrolled. In Umeå, more than 1300 patients with CRC have been recruited since the start in 2010.

The U-CAN exploration cohort (the Umeå Immune Profiling of Colorectal Cancer Project - UIP-CRC), comprises Stage I-IV CRC patients included in U-CAN between November 2015 and July 2017. For analyses of immune activity profiles by flow cytometry, a blood sample was collected in the morning on the day of surgery, and fresh tumour tissue was collected at the time of routine sampling at the Department of Clinical Pathology. A total of 153 CRC patients were included in U-CAN over the specified time-period. Of these, 69 Stage I-IV CRC patients were included in the UIP-CRC study as described previously^26^. Exclusions included pre-operatively irradiated rectal cancer patients, priority of routine diagnostic sampling, surgery out of laboratory hours, and lack of U-CAN referral. The UIP-CRC cohort has been well characterised regarding clinical and pathological characteristics and molecular determinants, including MSI status, DNA fragmentation analysis, *BRAF-* and *KRAS*-mutation status, and CMS subtype, as described previously^26^. Survival data were collected March 2022, and the median follow-up time from the time of surgery to death or end of follow-up was 60.8 months.

The U-CAN validation cohort comprises Stage I-IV CRC patients included in U-CAN between 2010 and 2014^46^. During this time, a total of 684 patients were included in U-CAN, of which 260 patients left a stool sample. Three of the patients included have since withdrawn their participation, leaving 257 CRC patients in the study. Tumour tissue was available from 187 patients operated on at Umeå University Hospital. For patients included in the study but operated on at another hospital (*n* = 59), tumour tissue was not accessible at this time. A tumour tissue microarray (TMA) was constructed in September 2020 from the patients with tumour tissue available at that time (*n* = 151), using the TMA GRAND Master instrument (3DHISTEC, Budapest, Hungary) to punch 1 mm cores from FFPE tumour tissue specimens. For this study, two cores taken at the tumour front from each patient were included and assessed. The U-CAN-validation cohort has been described according to clinical and pathological characteristics as well as molecular (*KRAS-*, *BRAF-*, and MSI status) characteristics, as described previously^46^. Survival data were collected in October 2021, and the median time from surgery to death or end of follow-up was 92.8 months.

The Ethics Committee of Umeå University/Regional Ethics Review Board approved all parts of the study protocol, including the procedure whereby patients gave written informed consent. The study was performed in accordance with the Declaration of Helsinki.

### CRP

Data on preoperative plasma CRP was collected by reviewing patient files. SIR was defined as a CRP level > 10 mg/l according to previous studies^7,47^. CRP data were accessible for 65 patients in the U-CAN exploration cohort and 182 patients in the U-CAN validation cohort. After reviewing patient files from healthcare centres and hospital clinics, all patients with a high CRP (> 10 mg/l), but with clinical evidence of infection (positive urine or blood culture), positive x-ray, or with clinical evidence of an inflammatory condition such as rheumatoid arthritis, were excluded. Patients taking antibiotics two weeks prior to blood sampling were also excluded, as well as patients with evidence of a second cancer diagnosis. After exclusions, CRP data were available from 57 patients in the U-CAN exploration cohort and 177 patients in the U-CAN validation cohort. Plasma CRP was assessed in the routine clinical setting, using a certified latex particle-enhanced immunologic turbidimetric assay (CRPL3 kit on a Cobas® instrument c501/701) by Roche Diagnostics.

### Immune activity profiles

In the U-CAN exploration cohort, immune activity profiles were analysed by flow cytometry from isolated mononuclear immune cells extracted from CRC tumour tissue and blood, as described previously^26^. Immune activity profiles were available from tumour tissue from 64 patients and blood samples from 49 patients. Lack of data on some parameters was due to lack of laboratory staff needed to handle fresh tissue or blood samples, and poor sample yield or quality. Briefly, mononuclear cells were gated in the FSC/SCC window and the following gating strategy was used to identify T-helper cells (CD3^+^/CD4^+^), cytotoxic T cells (CD3^+^/CD8^+^), monocytes/macrophages (CD14^+^), NK cells (CD56^+^/CD16^+^/CD3^−^), and B cells (CD19^+^). The identified cell populations were further gated using fluorescence minus one (FMO) control, for analyses of the expression of specific immune activity markers (CD28, CD69, PD-1, CTLA-4, NKG2D, CD80, CD86, CD163, HLA-DR, or PD-L1).

Non-fasting plasma samples collected at the time of diagnosis were available from 63 of the study patients. For analyses of systemic inflammatory markers, the Olink Immuno-oncology panel (v3.111) (Olink Biosciences, Uppsala, Sweden) was used, identifying 92 different proteins by Proximity Extension Assay technology. The analyses were run by SciLifeLab, Uppsala, Sweden. Data were presented as normalised protein expression (NPX) values on a log2-scale. The CMS classifier for RNA-sequencing data was applied according to consensus molecular subtype classification^12^. Due to lack of fresh frozen tumour tissue or poor RNA quality, 6 patients were excluded from the RNA sequencing analysis. By using the methodology for RNA sequencing based CMS classification, 54 of 63 samples could be classified as one of the four CMS types.

In the U-CAN validation cohort, immune cells were analysed using a multiplex immunohistochemistry approach (VECTRA^®^system for multispectral imaging), as described previously^48^. Briefly, TMA slides were immunohistochemically stained for T-helper cells (T-bet^+^), cytotoxic T cells (CD8^+^), regulatory T cells (FoxP3^+^), B cells (CD20^+^), macrophages (CD68^+^), and cytokeratin. The slides were imaged, and the images were imported to InForm^®^ software to train algorithms in the identification of tumour areas and immune cell phenotypes. For this study, immune cells were evaluated at the tumour front in 139 patients, in both the stromal area and the tumour epithelial area. Data were presented as the number of infiltrating immune cells per square millimeter. Patients with irradiated rectal cancer were excluded (*n* = 45).

## Statistics

IBM^®^ SPSS^®^Statistics 28 (SPSS Inc, Chicago, IL, USA) was used for statistical analyses. The Mann-Whitney U test was used to compare distributions of continuous variables between groups. Fischer´s exact test was used for comparisons of categorical variables. Differentially expressed proteins (DEPs) were determined using Welsh´s t-test and visualised in a volcano plot using R-package “ggplot2”^49^, in the statistical programming language R version 4.2.1 (R Core Team, Vienna, Austria)^50^. Correlations between continuous variables were analysed using the Spearman´s rank correlation test. Kaplan-Meier survival plots were used to estimate cancer-specific survival of Stage I-III CRC patients, where cancer-specific death was defined as death with disseminated or recurrent disease. Patients dying from postoperative complications within 90 days of surgery and patients not undergoing primary tumour surgery were excluded from the survival analysis. The log-rank test was used to estimate differences in 5-year cancer-specific survival to fulfil the assumption of proportional hazards (Fig. 3). A Cox proportional hazards model was applied for multivariable analysis of 5-year cancer specific survival. A *P* value < 0.05 was considered statistically significant.

## Electronic supplementary material

Below is the link to the electronic supplementary material.

Supplementary Material 1

## Data Availability

The data presented in this study are available from the corresponding author on reasonable request.

## Declarations

**Supplementary Information** accompanies this paper. Supplementary Table S1: Multivariable Cox regression analysis in Stage I-III patients from the U-CAN validation cohort.

## References

[1] Morgan, E. et al. Global burden of colorectal cancer in 2020 and 2040: incidence and mortality estimates from GLOBOCAN. *Gut* **72**, 338–344 (2023). 36604116 10.1136/gutjnl-2022-327736

[2] Lea, D., Håland, S., Hagland, H. R. & Søreide, K. Accuracy of TNM staging in colorectal cancer: a review of current culprits, the modern role of morphology and stepping-stones for improvements in the molecular era. *Scand. J. Gastroenterol.* **49**, 1153–1163 (2014). 25144865 10.3109/00365521.2014.950692

[3] Galon, J. et al. Type, density, and location of immune cells within human colorectal tumors predict clinical outcome. *Science* **313**, 1960–1964 (2006). 17008531 10.1126/science.1129139

[4] Wang, X., Duanmu, J., Fu, X., Li, T. & Jiang, Q. Analyzing and validating the prognostic value and mechanism of colon cancer immune microenvironment. *J. Transl Med.* **18**, 324 (2020). 32859214 10.1186/s12967-020-02491-w PMC7456375

[5] Park, J. H. et al. Systemic inflammation and outcome in 2295 patients with Stage I-III Colorectal Cancer from Scotland and Norway: first results from the ScotScan Colorectal Cancer Group. *Ann. Surg. Oncol.* **27**, 2784–2794 (2020). 32248375 10.1245/s10434-020-08268-1 PMC7334267

[6] Kersten, C. et al. Increased C-reactive protein implies a poorer stage-specific prognosis in colon cancer. *Acta Oncol.* **52**, 1691–1698 (2013). 24102179 10.3109/0284186X.2013.835494

[7] Køstner, A. H. et al. The prognostic role of systemic inflammation in patients undergoing resection of colorectal liver metastases: C-reactive protein (CRP) is a strong negative prognostic biomarker. *J. Surg. Oncol.* **114**, 895–899 (2016). 27696432 10.1002/jso.24415

[8] Woo, H. D., Kim, K. & Kim, J. Association between preoperative C-reactive protein level and colorectal cancer survival: a meta-analysis. *Cancer Causes Control*. **26**, 1661–1670 (2015). 26376895 10.1007/s10552-015-0663-8

[9] Thomsen, M. et al. Interleukin-6 and C-reactive protein as prognostic biomarkers in metastatic colorectal cancer. *Oncotarget* **7**, 75013–75022 (2016). 27738330 10.18632/oncotarget.12601 PMC5342719

[10] Casadei Gardini, A. et al. Prognostic role of serum concentrations of high-sensitivity C-reactive protein in patients with metastatic colorectal cancer: results from the ITACa trial. *Oncotarget* **7**, 10193–10202 (2016). 26848624 10.18632/oncotarget.7166 PMC4891113

[11] Ten Hoorn, S., de Back, T. R., Sommeijer, D. W. & Vermeulen, L. Clinical value of Consensus Molecular subtypes in Colorectal Cancer: a systematic review and Meta-analysis. *J. Natl. Cancer Inst.* **114**, 503–516 (2022). 34077519 10.1093/jnci/djab106 PMC9002278

[12] Guinney, J. et al. The consensus molecular subtypes of colorectal cancer. *Nat. Med.* **21**, 1350–1356 (2015). 26457759 10.1038/nm.3967 PMC4636487

[13] Guastadisegni, C., Colafranceschi, M., Ottini, L. & Dogliotti, E. Microsatellite instability as a marker of prognosis and response to therapy: a meta-analysis of colorectal cancer survival data. *Eur. J. Cancer*. **46**, 2788–2798 (2010). 20627535 10.1016/j.ejca.2010.05.009

[14] Gkekas, I. et al. Deficient mismatch repair as a prognostic marker in stage II colon cancer patients. *Eur. J. Surg. Oncol.* **45**, 1854–1861 (2019). 31186203 10.1016/j.ejso.2019.05.023

[15] Popat, S., Hubner, R. & Houlston, R. S. Systematic review of microsatellite instability and colorectal cancer prognosis. *J. Clin. Oncol.* **23**, 609–618 (2005). 15659508 10.1200/JCO.2005.01.086

[16] Kuznetsova, O. et al. Prognostic and predictive role of immune microenvironment in colorectal cancer. *World J. Gastrointest. Oncol.* **16**, 643–652 (2024). 38577454 10.4251/wjgo.v16.i3.643 PMC10989368

[17] Yamauchi, M. et al. Colorectal cancer: a tale of two sides or a continuum? *Gut* **61**, 794–797 (2012). 22490520 10.1136/gutjnl-2012-302014 PMC3345045

[18] Söreide, K., Janssen, E. A., Söiland, H., Körner, H. & Baak, J. P. Microsatellite instability in colorectal cancer. *Br. J. Surg.* **93**, 395–406 (2006). 16555243 10.1002/bjs.5328

[19] Nosho, K. et al. Tumour-infiltrating T-cell subsets, molecular changes in colorectal cancer, and prognosis: cohort study and literature review. *J. Pathol.* **222**, 350–366 (2010). 20927778 10.1002/path.2774 PMC3033700

[20] Song, J. et al. The impact of molecular profile on the lymphatic spread pattern in stage III colon cancer. *Cancer Sci.* **112**, 1545–1555 (2021). 33484192 10.1111/cas.14819 PMC8019193

[21] He, W. Z. et al. Systemic neutrophil lymphocyte ratio and mismatch repair status in colorectal cancer patients: correlation and prognostic value. *J. Cancer*. **9**, 3093–3100 (2018). 30210632 10.7150/jca.26669 PMC6134814

[22] Li, J. et al. Systemic inflammatory markers of Resectable Colorectal Cancer patients with different mismatch repair gene status. *Cancer Manag Res.* **13**, 2925–2935 (2021). 33833576 10.2147/CMAR.S298885 PMC8019618

[23] Park, J. H. et al. Mismatch repair status in patients with primary operable colorectal cancer: associations with the local and systemic tumour environment. *Br. J. Cancer*. **114**, 562–570 (2016). 26859693 10.1038/bjc.2016.17 PMC4782207

[24] Baeten, C. I., Castermans, K., Hillen, H. F. & Griffioen, A. W. Proliferating endothelial cells and leukocyte infiltration as prognostic markers in colorectal cancer. *Clin. Gastroenterol. Hepatol.* **4**, 1351–1357 (2006). 17059898 10.1016/j.cgh.2006.08.005

[25] Gunnarsson, U. et al. Association between local immune cell infiltration, mismatch repair status and systemic inflammatory response in colorectal cancer. *J. Transl Med.* **18**, 178 (2020). 32316975 10.1186/s12967-020-02336-6 PMC7175507

[26] Li, X. et al. A detailed Flow Cytometric Analysis of Immune Activity profiles in Molecular subtypes of Colorectal Cancer. *Cancers (Basel)* 12 (2020). 10.3390/cancers12113440 PMC7699331 33228141

[27] Angelova, M. et al. Characterization of the immunophenotypes and antigenomes of colorectal cancers reveals distinct tumor escape mechanisms and novel targets for immunotherapy. *Genome Biol.* **16**, 64 (2015). 25853550 10.1186/s13059-015-0620-6 PMC4377852

[28] Domingo, E. et al. Somatic POLE proofreading domain mutation, immune response, and prognosis in colorectal cancer: a retrospective, pooled biomarker study. *Lancet Gastroenterol. Hepatol.* **1**, 207–216 (2016). 28404093 10.1016/S2468-1253(16)30014-0

[29] Giannakis, M. et al. Genomic correlates of Immune-Cell infiltrates in Colorectal Carcinoma. *Cell. Rep.* **15**, 857–865 (2016). 27149842 10.1016/j.celrep.2016.03.075 PMC4850357

[30] van den Bulk, J. et al. Neoantigen-specific immunity in low mutation burden colorectal cancers of the consensus molecular subtype 4. *Genome Med.* **11**, 87 (2019). 31888734 10.1186/s13073-019-0697-8 PMC6938004

[31] Kostner, A. H. et al. Systemic inflammation associates with a myeloid Inflamed Tumor Microenvironment in primary resected Colon cancer-May Cold tumors simply be too hot? *Front. Immunol.* **12**, 716342 (2021). 34531864 10.3389/fimmu.2021.716342 PMC8438238

[32] Edin, S. et al. The distribution of macrophages with a M1 or M2 phenotype in relation to prognosis and the molecular characteristics of colorectal cancer. *PLoS One*. **7**, e47045 (2012). 23077543 10.1371/journal.pone.0047045 PMC3471949

[33] Chung, Y. C. & Chang, Y. F. Serum interleukin-6 levels reflect the disease status of colorectal cancer. *J. Surg. Oncol.* **83**, 222–226 (2003). 12884234 10.1002/jso.10269

[34] Nikiteas, N. I. et al. Serum IL-6, TNFalpha and CRP levels in Greek colorectal cancer patients: prognostic implications. *World J. Gastroenterol.* **11**, 1639–1643 (2005). 15786541 10.3748/wjg.v11.i11.1639 PMC4305945

[35] Guthrie, G. J., Roxburgh, C. S., Horgan, P. G. & McMillan, D. C. Does interleukin-6 link explain the link between tumour necrosis, local and systemic inflammatory responses and outcome in patients with colorectal cancer? *Cancer Treat. Rev.* **39**, 89–96 (2013). 22858249 10.1016/j.ctrv.2012.07.003

[36] Belluco, C. et al. Interleukin-6 blood level is associated with circulating carcinoembryonic antigen and prognosis in patients with colorectal cancer. *Ann. Surg. Oncol.* **7**, 133–138 (2000). 10761792 10.1007/s10434-000-0133-7

[37] Yeh, K. Y. et al. Analysis of the effect of serum interleukin-6 (IL-6) and soluble IL-6 receptor levels on survival of patients with colorectal cancer. *Jpn J. Clin. Oncol.* **40**, 580–587 (2010). 20194250 10.1093/jjco/hyq010

[38] Imamura, Y. et al. Specific mutations in KRAS codons 12 and 13, and patient prognosis in 1075 BRAF wild-type colorectal cancers. *Clin. Cancer Res.* **18**, 4753–4763 (2012). 22753589 10.1158/1078-0432.CCR-11-3210 PMC3624899

[39] Modest, D. P. et al. Outcome according to KRAS-, NRAS- and BRAF-mutation as well as KRAS mutation variants: pooled analysis of five randomized trials in metastatic colorectal cancer by the AIO colorectal cancer study group. *Ann. Oncol.* **27**, 1746–1753 (2016). 27358379 10.1093/annonc/mdw261 PMC4999563

[40] Taieb, J. et al. Different prognostic values of KRAS exon 2 submutations and BRAF V600E mutation in microsatellite stable (MSS) and unstable (MSI) stage III colon cancer: an ACCENT/IDEA pooled analysis of seven trials. *Ann. Oncol.* **34**, 1025–1034 (2023). 37619846 10.1016/j.annonc.2023.08.006 PMC10938565

[41] Lochhead, P. et al. Microsatellite instability and BRAF mutation testing in colorectal cancer prognostication. *J. Natl. Cancer Inst.* **105**, 1151–1156 (2013). 23878352 10.1093/jnci/djt173 PMC3735463

[42] Liu, J. et al. Immune landscape and prognostic immune-related genes in KRAS-mutant colorectal cancer patients. *J. Transl Med.* **19**, 27 (2021). 33413474 10.1186/s12967-020-02638-9 PMC7789428

[43] Maker, A. V. et al. Genetic evidence that intratumoral T-cell proliferation and activation are associated with recurrence and survival in patients with resected colorectal liver metastases. *Cancer Immunol. Res.* **3**, 380–388 (2015). 25600439 10.1158/2326-6066.CIR-14-0212 PMC4390462

[44] Mori, K. et al. Systemic analysis of predictive biomarkers for recurrence in Colorectal Cancer patients treated with curative surgery. *Dig. Dis. Sci.* **60**, 2477–2487 (2015). 25840921 10.1007/s10620-015-3648-2

[45] Glimelius, B. et al. U-CAN: a prospective longitudinal collection of biomaterials and clinical information from adult cancer patients in Sweden. *Acta Oncol.* **57**, 187–194 (2018). 28631533 10.1080/0284186X.2017.1337926

[46] Löwenmark, T. et al. Tumour Colonisation of Parvimonas micra is Associated with decreased survival in Colorectal Cancer patients. *Cancers (Basel)* 14 (2022). 10.3390/cancers14235937 PMC9736682 36497419

[47] McMillan, D. C. The systemic inflammation-based Glasgow Prognostic Score: a decade of experience in patients with cancer. *Cancer Treat. Rev.* **39**, 534–540 (2013). 10.1016/j.ctrv.2012.08.003 22995477

[48] Edin, S. et al. Opposing roles by KRAS and BRAF mutation on immune cell infiltration in colorectal cancer - possible implications for immunotherapy. *Br. J. Cancer* (2023). 10.1038/s41416-023-02483-9 PMC10781968 38040818

[49] Wickham, H. *ggplot2: Elegant Graphics for Data Analysis* (Springer-, 2016).

[50] R Core Team. R: A language and environment for statistical computing. *R foundation for Statistical Computing, Vienna, Austria*, (2022). https://www.R-project.org/

