# Supplementary material for: Systemic inflammatory response in colorectal cancer is associated with tumour mismatch repair and impaired survival
Source: Sci Rep. 2024 Nov 29;14:29738. doi: 10.1038/s41598-024-80803-6 (PMC11607405; doi:10.1038/s41598-024-80803-6)
Supplement: Supplementary file 1 — Supplementary Material 1 [file 41598_2024_80803_MOESM1_ESM.pdf]

Supplementary Information

Supplementary Table S1. Multivariable Cox regression analysis in stage I-III patients from the U-CAN validation cohort.

Supplementary Table S1. Multivariable Cox regression analyses in stage I-III patients from the U-CAN validation cohort.

	Univariable			Multivariable		
	HR	95% CI	*P* value	HR	95%CI	*P* value
Age	0.94	0.89 - 0.99	0.023	0.90	0.84 - 0.98	0.009
Sex						
Female	1	-	-	1	-	-
Male	0.94	0.30 - 2.92	0.917	2.65	0.71 - 9.87	0.145
Tumour site						
Right colon	1	-	-	1	-	-
Left colon	0.92	0.15 - 5.53	0.931	0.68	0.10 - 4.41	0.685
Rectum	1.33	0.34 - 5.14	0.681	3.55	0.78 - 16.1	0.100
Stage						
I-II	1	-	-	1	-	-
III	7.05	1.91 - 26.0	0.003	7.67	1.75 - 33.7	0.007
CRP						
Low	1	-	-	1	-	-
High	4.11	1.30 – 13.0	0.016	12.8	2.60 – 63.0	0.002

Abbreviations: HR, hazard ratio; CI, confidence interval.
